# Interferon-induced transmembrane protein 1 (*IFITM1*) overexpression enhances the aggressive phenotype of SUM149 inflammatory breast cancer cells in a signal transducer and activator of transcription 2 (STAT2)-dependent manner

**DOI:** 10.1186/s13058-016-0683-7

**Published:** 2016-02-20

**Authors:** Joshua Ogony, Hye Joung Choi, Asona Lui, Massimo Cristofanilli, Joan Lewis-Wambi

**Affiliations:** Department of Cancer Biology, University of Kansas Medical Center, Kansas City, KS USA; Department of Molecular and Integrative Physiology, University of Kansas Medical Center, Kansas City, KS USA; Department of Medical Oncology, Northwestern University, Chicago, IL USA

**Keywords:** Inflammatory breast cancer, IFITM1, JAK/STAT signaling, Interferon-stimulated genes, BRG1

## Abstract

**Background:**

Inflammatory breast cancer (IBC) is a very aggressive and lethal subtype of breast cancer that accounts for about 4 % of all breast cancers diagnosed in the United States. Despite the efforts of several investigators to identify the molecular factors driving the aggressive phenotype of IBC, a great deal is still unknown about the molecular underpinnings of the disease. In the present study, we investigated the role of interferon-induced transmembrane protein 1 (*IFITM1*), a well-known interferon-stimulated gene (ISG), in promoting the aggressiveness of SUM149 IBC cells.

**Methods:**

Western blot and real-time polymerase chain reaction analyses were performed to assess the protein and messenger RNA (mRNA) levels of *IFITM1* and other ISGs in three IBC cell lines: SUM149, MDA-IBC-3, and SUM190. *IFITM1* expression and cellular localization were assessed by using immunofluorescence, while the tumorigenic potential was assessed by performing cell migration, invasion, and colony formation assays. Small interfering RNA and short hairpin RNA knockdowns, enzyme-linked immunosorbent assays, and luciferase assays were performed to determine the functional significance of IFITM1 and signal transducers and activators of transcription 1 and 2 (STAT1/2) in SUM149 cells.

**Results:**

We found that *IFITM1* was constitutively overexpressed at the mRNA and protein levels in triple-negative SUM149 IBC cells, but that it was not expressed in SUM190 and MDA-IBC-3 IBC cells, and that suppression of IFITM1 or blockade of the IFNα signaling pathway significantly reduced the aggressive phenotype of SUM149 cells. Additionally, we found that knockdown of STAT2 abolished IFITM1 expression and IFITM1 promoter activity in SUM149 cells and that loss of STAT2 significantly inhibited the ability of SUM149 cells to proliferate, migrate, invade, and form 2-D colonies. Notably, we found that STAT2-mediated activation of IFITM1 was particularly dependent on the chromatin remodeler brahma-related gene 1 (BRG1), which was significantly elevated in SUM149 cells compared with SUM190 and MDA-IBC-3 cells.

**Conclusions:**

These findings indicate that overexpression of *IFITM1* enhances the aggressive phenotype of triple-negative SUM149 IBC cells and that this effect is dependent on STAT2/BRG1 interaction. Further studies are necessary to explore the potential of *IFITM1* as a novel therapeutic target and prognostic marker for some subtypes of IBCs.

**Electronic supplementary material:**

The online version of this article (doi:10.1186/s13058-016-0683-7) contains supplementary material, which is available to authorized users.

## Background

Inflammatory breast cancer (IBC) is one of the most aggressive and lethal subtypes of human breast cancer, accounting for about 4 % of all breast cancer cases diagnosed in the United States. A recent study published by Fouad et al. [1] showed that the 5-year overall survival for patients diagnosed with stage IV IBC is significantly lower than that of patients diagnosed with stage IV non-IBC, emphasizing the lethality of IBC [2–5]. In the last decade, remarkable progress has been made toward genomic profiling of IBC, leading to identification of molecular alterations commonly found in these tumors. The most notable alterations that have been reported include lack of estrogen and progesterone receptors (ER−/PR−); overexpression of epidermal growth factor receptor, ERBB2/human epidermal growth factor receptor 2 (HER2), E-cadherin, eIF4GI, chemokines, and chemokine receptors; dysfunction of mucin 1; high proliferation; tumor protein 53 mutations; and elevated angiogenesis [6]. Despite these efforts, however, there is still a great deal that is not known about the biology of IBC or the factors that drive its aggressive phenotype.

A recent study by the international IBC consortium reported that the interferon alpha (IFNα) signaling pathway was significantly upregulated in IBC [7]. Interferons (IFNs) are cytokines that affect biological responses through the Janus kinase/signal transducer and activator of transcription (JAK-STAT) signaling pathway. This pathway involves IFNs, which, acting as ligands, bind their corresponding receptors [interferon (alpha, beta and omega) receptor (IFNAR)/interferon gamma receptor], resulting in the phosphorylation and activation of STAT1 and STAT2 and subsequent transcription of interferon-stimulated genes (ISGs), which include *STAT1*, *STAT2*, phospholipid scramblase 1 (*PLSCR1*), interferon-induced transmembrane protein 1 (*IFITM1*), interferon-inducible protein 27 (*IFI27*), interferon-induced protein with tetratricopeptide repeats 1 (*IFIT1*), and many others [8]. The transcription of these ISGs requires the remodeling of their promoter regions to increase accessibility to transcription factors. Brahma-related gene 1 (BRG1), which is the adenosine triphosphate (ATP) subunit of the brahma-associated factor (BAF) complex, is responsible for the remodeling of the promoter region of many ISGs and is recruited to its site of action by STAT2 [9]. Several studies have suggested that altered type I IFNα/IFNβ signaling, resulting in increased expression of ISGs, might play a role in tumorigenesis and contribute to poor patient prognosis [10–13]. Indeed, increased expression of *IFITM1*, a well-known ISG, has been shown to correlate with disease progression, resistance to endocrine therapy and chemotherapy, and worse overall prognosis in patients with gastrointestinal, colorectal, and breast cancers [14, 15].

*IFITM1* is a member of the IFITM protein family whose expression is strongly induced by type I IFNs [16]. It was initially identified as a leukocyte antigen that is part of a membrane complex involved in the transduction of antiproliferative and homotypic cell adhesion signals in lymphocytes [17]. Most recently, however, there has been evidence to suggest that IFITM1 might also play a role in tumorigenesis. *IFITM1* has been shown to be overexpressed in several types of cancers, including colorectal, gastrointestinal, head and neck, and breast cancers, and its overexpression positively correlates with tumor progression and increased invasiveness [14, 18–21]. We hypothesized that hyperactivation of the IFNα signaling pathway drives *IFITM1* overexpression, which enhances the aggressive phenotype of IBC cells.

In this study, we measured *IFITM1* expression in three IBC cell lines—SUM149, SUM190, and MDA-IBC-3—and in a non-IBC breast cancer cell line, MCF-7. We found that IFITM1 was highly expressed in SUM149 cells, which are ER−/PR−/HER2−, but not expressed in HER2-overexpressing SUM190 and MDA-IBC-3 cells or ER+/PR+ MCF-7 cells. We also found that *IFITM1* overexpression promoted—whereas its knockdown inhibited—proliferation, migration, invasion, and tumorigenicity in SUM149 cells. Additionally, we determined that blockade of IFNα signaling using a neutralizing antibody against its receptor, IFNAR1/2, or knockdown of STAT2 and the chromatin remodeling protein BRG1, dramatically reduced *IFITM1* expression and the tumorigenic potential of SUM149 cells. These findings suggest a critical role for IFNα signaling and STAT2-mediated activation of *IFITM1* in promoting the aggressiveness of triple-negative SUM149 IBC cells; however, additional studies need to be performed in other triple-negative inflammatory breast cancer (TNIBC) cell lines as well as in IBC tumors to validate the biological and clinical significance of these findings in IBC.

## Methods

### Reagents

Ham’s F-12 (1×) nutrient mixture (catalogue number 11765-054), RPMI 1640 medium (catalogue number 11875-093), fetal bovine serum (FBS; catalogue number 16000-044), antibiotic/antimycotic solution (containing 10,000 U/ml penicillin, 10 mg/ml streptomycin, and 25 μg/ml Fungizone®), minimum essential medium nonessential amino acids, l-glutamine, and TrypLE (containing trypsin and ethylenediaminetetraacetic acid) were obtained from Life Technologies (Grand Island, NY, USA). Insulin (bovine pancreas), anti-β-actin, and hydrocortisone were obtained from Sigma-Aldrich (St. Louis, MO, USA). Anti-IFITM1, anti-STAT1, anti-STAT2, anti-BRG1, anti-p-STAT2 (Tyr690), anti-interferon regulatory factor (IRF)-7, anti-IFNα, anti-p21, anti-cyclin D1, and anti-cyclin E antibodies were purchased from Santa Cruz Biotechnology (Santa Cruz, CA, USA), and rabbit polyclonal and mouse monoclonal secondary antibodies and anti-p-STAT1 (Tyr701) were purchased from Cell Signaling Technology (Danvers, MA, USA). IFITM1 promoter constructs were kindly provided by Dr. Yeon-Su Lee from the Cancer Genomics Branch, National Cancer Center, Goyang-si, South Korea.

### Cell lines and culture conditions

Experiments were performed using the IBC cell lines SUM149, SUM190, and MDA-IBC-3 and a non-IBC breast cancer cell line, MCF-7. SUM149 and SUM190 cells were obtained from Dr. Massimo Cristofanilli (Northwestern University, Chicago, IL, USA), who purchased them from Asterand Bioscience (Detroit, MI, USA). MDA-IBC-3 cells were developed by Dr. Wendy Woodward (The University of Texas MD Anderson Cancer Center, Houston, TX, USA) and were provided to us by Dr. Massimo Cristofanilli (Northwestern University, Chicago IL). The IBC cells were maintained in Ham’s F-12 nutrient mixture supplemented with 10 % FBS, 5 μg/ml insulin, 1 μg/ml hydrocortisone, and 100 U/ml antibiotic-antimycotic. The ER+, hormone-dependent human breast cancer cell line MCF-7 was obtained from the American Type Culture Collection (Manassas, VA, USA) and was maintained in full serum medium composed of RPMI 1640 medium, 10 % FBS, 2 mM glutamine, penicillin at 100 U/ml, streptomycin at 100 μg/ml, 1× nonessential amino acids (Life Technologies), and bovine insulin at 6 ng/ml (Sigma-Aldrich, St Louis, MO). The cells were passaged twice weekly, with media changed every other day, and they were cultured at 37 °C in a 5 % CO_2_ atmosphere. The cells were plated and incubated overnight for attachment before the treatment protocols were begun.

### Anchorage-independent growth

Anchorage-independent growth was performed in soft agar for SUM149 and SUM190 cells. The experiments were performed in 6-well plates as per the protocol published by Debeb et al. [22]. Briefly, the base layer was made of 2 ml of medium containing 1 % FBS and 0.5 % agarose (A9539-100G; Sigma-Aldrich). A quantity of 5 × 10^4^ cells was layered onto the base in 2 ml of medium containing 1 % FBS and 0.35 % agarose. The plates were briefly cooled at 4 °C so that agarose could solidify before the growth medium was added, and the plates were kept in the 37 °C incubator for 21 days. Images of the colonies were captured using the ChemiDoc™ XRS System equipped with Image Lab™ software (Bio-Rad Laboratories, Hercules, CA, USA), and using a phase-contrast microscope equipped with an Olympus camera (Olympus America, Center Valley, PA, USA).

### 2-D colony formation

SUM149 cells were plated at low density in 6-well plates and cultured for 7 days, with media changed every other day. At the end of the 7 days, the colonies were stained with 5 % crystal violet for 5 minutes and then washed. The images of the colonies were captured using the ChemiDoc™ XRS System equipped with Image Lab™ software.

### Wound-healing assay

SUM149 and SUM190 cells were seeded at a density of 3.0 × 10^5^ cells per well in 6-well culture plates overnight so that the cells would attach. A single wound was made on the plates for each cell line by scratching the attached cells using a 200-μl sterile pipette tip. The plates were washed with complete medium to remove cellular debris from the scraped surface. The images of the cells were taken immediately and after 24, 48, and 72 h using a phase-contrast microscope.

### Cell invasion assay

Cell invasion measurements were performed using the Chemicon 24-well QCM ECMatrix Cell Invasion Assay (ECM 554; EMD Millipore, Billerica, MA, USA) according to the manufacturer’s protocol. Briefly, IBC cells (1 × 10^5^) in 250 μl of serum-free medium were added to each insert, and 500 μl of media with or without a chemoattractant (10 % FBS) was added to the lower chamber. The plates were returned to the incubator at 37 °C in a 5 % CO_2_ atmosphere for 48 h. The media with noninvading cells were removed from the inserts, and the inserts were placed in cell detachment medium and incubated for 30 minutes at 37 °C to dislodge the invaded cells. The invaded cells were lysed in the presence of Molecular Probes CyQUANT GR Dye (Life Technologies, Eugene, OR, USA) for 15 minutes at room temperature, and the fluorescence of 200-μl aliquots in 96-well plates was measured using a fluorescence plate reader with a 480/520-nm filter set. For a second set of inserts, the migrated cells were stained with crystal violet and the images were captured with a phase-contrast microscope equipped with an Olympus camera.

### MTT assay

Proliferation of IBC cells was determined by using a 3-(4,5-dimethylthiazol-2-yl)-2,5-diphenyltetrazolium bromide (MTT) assay. SUM149 and SUM190 cells were seeded onto 24-well plates at a density of 2.5 × 10^4^ cells per well in cell culture media and incubated overnight for attachment, followed by treatment protocols. A 50-μl MTT solution (5 mg/ml) was added to each well at a final concentration of 500 μg/ml, and the mixture was further incubated for 3 h at 37 °C. A quantity of 500 μl of the solubilizing solution (dimethyl sulfoxide/ethanol at 1:1 vol/vol) was added to each well and shaken to dissolve the crystals. The absorbance was read with a VMax microplate reader (Molecular Devices, Sunnyvale, CA, USA) at 570 nM, and the relative cell proliferation was expressed as a percentage of the control.

### Western blotting

The cells were detached using a cell scraper and pelleted in 1.5-ml microcentrifuge tubes, then lysed with cell lysis buffer [150 mM NaCl, 1.0 % IGEPAL® CA-630 (Sigma-Aldrich), 0.5 % sodium deoxycholate, 0.1 % sodium dodecyl sulfate (SDS), 50 mM Tris, pH 8.0; Thermo Fisher Radioimmunoprecipitation Assay (RIPA) Lysis and Extraction Buffer, catalogue number 89901, Pierce Biotechnology/Thermo Fisher Scientific, Rockford, IL, USA] containing 1 % protease inhibitor and phosphatase inhibitor cocktail. The protein concentration was determined using a Bio-Rad protein assay (Bio-Rad Laboratories). Proteins (30 μg) from each sample were separated by 4-12 % SDS–polyacrylamide gel electrophoresis and electrically transferred to a polyvinylidene difluoride membrane (Bio-Rad Laboratories). The membranes were blocked in 5 % nonfat milk in Tris-buffered saline and Tween 20 (TBS-T) for 1 h and then incubated with primary antibodies (Santa Cruz Biotechnology) at 4 °C overnight. The membranes were washed three times for 10 minutes in TBS-T and incubated with corresponding secondary antibodies (Cell Signaling Technology) conjugated to horseradish peroxidase anti-rabbit (or anti-mouse) immunoglobulin G (IgG) in 5 % nonfat milk in TBS-T for 1 h at room temperature, followed by three washes for 10 minutes. Immunoreactivity was detected by enhanced chemiluminescence Western blotting detection reagents (Amersham ECL Plus; GE Healthcare Life Sciences, Piscataway, NJ, USA). The Western blot quantitation was performed using ImageJ software (http://rsb.info.nih.gov/ij/download.html; National Institutes of Health, Bethesda, MD, USA).

### Small interfering RNA transfection

SUM149 cells were transfected with pooled small interfering RNAs (siRNAs) targeting IFITM1, STAT1, STAT2, BRG1, IFNα2, and IRF7. All of the pooled siRNAs contained a mixture of three target-specific 20- to 25-nt siRNAs. For IFITM1 knockdown, the individual siRNAs used were siRNA 1 (sc-44549A), siRNA 2 (sc-44549B), and siRNA 3 (sc-44549). For STAT1 and STAT2 knockdown, both individual and pooled siRNAs were used. The individual siRNAs for STAT1 were siRNA 1 (sc-44123A), siRNA 2 (sc-44123B), and siRNA 3 (sc-44123), and the individual siRNAs for STAT2 were siRNA 1 (sc-29492A), siRNA 2 (sc-29492B), and siRNA 3 (sc-29492). siBRG1 (sc-29827), siIRF7 (sc-38011), small interfering interferon α2 (siIFNα2; sc-63324), and scrambled RNA (siCon; sc-37007) were purchased from Santa Cruz Biotechnology. The transfection reagent used was Lipofectamine 2000™ from Life Technologies (Carlsbad, CA, USA). IBC cells were seeded overnight and then transfected at a density of 50–60 % confluence with 60–100 nM of targeted siRNAs or siCon. Transfected cells were maintained in culture for 24–72 h before being harvested and further analyzed. We should note that the knockdown efficiency of individual siRNAs targeting IFITM1, STAT1, and STAT2 was not statistically significantly different from that of the pooled siRNAs; hence, the pooled siRNAs were used for the functional studies.

### Short hairpin RNA transfection

SUM149 cells were transfected with IFITM1 short hairpin RNA (shRNA) plasmid (h) (shIFITM1; sc-44549-SH) or control shRNA (shControl; sc-108060) plasmid, both of which were purchased from Santa Cruz Biotechnology. The IFITM1 shRNA plasmid was a pool of three different shRNA plasmids: SHA, SHB, and SHC. sc-44549-SHA: hairpin sequence: GATCCCACACTTCTCAAACCTTCATTCAAG AGATGAAGGTTTGAGAAGTGTGTTTTT; corresponding siRNA sequences (sc-44549A): sense: CACACUUCUCAAACCUUCAtt; antisense: UGAAGGUUUGAGAAGUGUGtt; sc-44549-SHB: hairpin sequence: GATCCCTGTGACAGTCTACCATATTTCAAGAGAATA TGGTAGACTGTCACAGTTTTT; corresponding siRNA sequences (sc-44549B): sense: CUGUGACAGUCUACCAUAUtt; antisense: AUAUGGUAGACUGUCACAGtt; sc-44549-SHC: hairpin sequence: GATCCCTGTCTACAGTGTCATTCATTCAAGAGATGAATGACA CTGTAGACAGTTTTT; corresponding siRNA sequences (sc-44549C): sense: CUGUCUACAGUGUCAUUCAtt; antisense: UGAAUGACACUGUAGACAGtt. SUM149 cells were seeded into 6-well plates, and at 60–70 % confluence they were transfected with 6–10 μg of shIFITM1 or shControl plasmid using Lipofectamine 2000™ reagent according to the manufacturer’s instructions. The transfected cells were incubated for 24–72 h, and the knockdown was confirmed by Western blot analysis and real-time polymerase chain reaction (RT-PCR).

### RNA isolation and RT-PCR analysis

Total RNA was isolated from cultured cells using a QIAGEN RNeasy Mini Kit Qiagen (catalogue number 74104; QIAGEN, Valencia, CA, USA) according to the manufacturer’s protocol. First-strand complementary DNA (cDNA) synthesis was performed using 2.5 μg of total RNA using Invitrogen SuperScript Reverse Transcriptase (Thermo Fisher Scientific, Carlsbad, CA, USA). cDNA was amplified in a 25-μl PCR mixture containing 1 μl of deoxynucleotide triphosphates, 1× PCR buffer, 2.5 mM MgCl_2_, and 1 U of DNA Taq polymerase (Promega, Madison, WI, USA) with 25 pmol of primers specific for human IFITM1, which were obtained from Integrated DNA Technologies (Coralville, IA, USA) (sense: 5′-GGATTTCGGCTTGTCCCGAG-3′, antisense: 5′-CCATGTGGAAGGGAGG GCTC-3′), STAT1 (sense: 5′-GGCACCAGAACGAATGAGGG-3′, antisense: 5′- CCATCGTGCACATGGTGGAG-3′), PLSCR1 (sense: 5′-CATTCACCGGGCTCTCTAC-3′, antisense: 5′-GGCAGCTGGGCAATCTTGCA-3′), STAT2 (sense: 5′-GCAGCACAAT TTG GGAA-3′, antisense: 5′-ACAGGTGTTTCGAGAACTGGC-3′), IRF9 (sense: 5′-TTCTGTCC CTGGTGTAGAGCCT-3′, antisense: 5′- TTTCAGGACACGATTATCACGG-3′), IRF7 sense: 5′-GAGCCCTTACCTCCCCTGTTAT-3′, antisense: 5′-CCACTGCAGCCCCTCATAG-3′, IFI27 (sense: 5′- GCCTCTGGCTCTGCCGTAGTT-3′, antisense: 5′-ATGGAGGACGAGGCGATTCC-3′), IFIT1 (sense: 5′-TCTCAGAGGAGCCTGGCTAA-3′, antisense: 5′-CCAGACTATCCTT GACCTGATGA-3′), OAS1 (sense: 5′-TGAGGTCCAGGCTCCACGCT-3′, antisense: 5′-GCAGGTCGGTGCACTCCTCG-3′). The PCR experiment was performed according to the manufacturer’s protocol for Applied Biosystems Power SYBR® Green PCR Master Mix and RT-PCR (PN 4367218; Life Technologies, Carlsbad, CA, USA) as follows: enzyme activation step: 10-minute hold at 95 °C for AmpliTaq Gold® enzyme activation, followed by PCR amplification steps (40 cycles); and denaturation step: 15 seconds at 95 °C, annealing/extension step: 60 seconds at 60 °C. Pumilio RNA-binding family member 1 (PUM1) was used as the internal control (sense: 5′-TCACCGAGGCCCCTCTGAACCCTA-3′; antisense: 5′-GGCAGTAATCTCCTTCTGCATCC T-3′). The reproducibility of the quantitative measurements was evaluated by three independent cDNA syntheses and PCR amplification from each preparation of messenger RNA (mRNA). The relative mRNA expression level was determined as a ratio of the signal intensity to that of PUM1.

### Cell-cycle analysis

SUM149 cells were seeded at a density of 3.0 × 10^5^ cells per well in 6-well culture plates overnight so that cells could attach. The cells were transfected with small interfering IFITM1 or control siRNA for 24 h. At the end of the transfection period, the cells were harvested by trypsinization and washed once with phosphate-buffered saline (PBS), pH 7.4. The cells were fixed in 100 % ice-cold ethanol for 24 h and stained with 50 μl/ml of 2 mg/ml propidium iodide stock with 10 μl/ml of RNase A and incubated for 30 minutes at room temperature. The DNA contents of 5 × 10^5^ cells were determined using a BD LSR II flow cytometer (BD Biosciences, San Jose, CA, USA). The flow cytometry data were analyzed using FlowJo software (Tree Star, Ashland, OR, USA) to determine the percentages of the cells at each phase of the cell cycle. Three separate experiments were performed in triplicate, and IFITM1 knockdown was confirmed by Western blot analysis after each transfection.

### Immunofluorescence microscopy

SUM149 and SUM190 IBC cells were plated onto chambered slides and, after overnight incubation for attachment, were treated with or without IFN for a further 24 h. The cells were washed in PBS and fixed with 100 % methanol for 10 minutes, and then washed three times for 10 minutes each before being permeabilized with 0.1 % Triton X-100 in PBS for 10 minutes, followed by three washes for 5 minutes and then blocking with 5 % normal horse serum for 1 h. The cells were then incubated with anti-IFITM1 primary antibody overnight, after which they were washed three times in PBS for 10 minutes, followed by staining with fluorescein isothiocyanate–conjugated goat anti-mouse IgG secondary antibody (4 μg/ml) for 1 h. The coverslips were mounted on glass slides with VECTASHIELD Mounting Medium (Vector Laboratories, Burlingame, CA, USA) containing 4′,6-diamidino-2-phenylindole for nuclear counterstaining, and samples were incubated at room temperature for 24 h before being analyzed using a Leica confocal microscope equipped with Leica Application Suite Advanced Fluorescence Lite 2.6.0 Build 7266 software (Leica Biosystems, Buffalo Grove, IL, USA).

### Enzyme-linked immunosorbent assay

Human IFNα levels were measured by enzyme-linked immunosorbent assay (ELISA; PBL Interferon Source; PBL Assay Science, Piscataway, NJ, USA). A quantity of 5 × 10^5^ SUM149, SUM190, and MCF-7 cells were seeded into 6-well plates and incubated at 37 °C overnight so that the cells would attach. The cells were then treated with 100 U/ml human recombinant IFNα or transfected with siIRF7 and siIFNα for 48 h. Cells and supernatants were harvested and kept at −80 °C until analysis. Protein was extracted by sonication in RIPA buffer supplemented with protease and phosphatase inhibitors. Supernatants and lysates were purified by centrifugation and analyzed for the presence of IFNα according to the manufacturer’s instructions.

### Interferon α receptor neutralization

SUM149 cells were treated with 5 μg/ml anti-IFNAR1/2 mouse anti-human IFNα/β receptor monoclonal antibody (catalogue number MAB1155; EMD Millipore) for 24 h. The cells were harvested using a cell scraper and then processed for Western blot analysis.

### IFITM1 luciferase promoter assay

SUM149 and SUM190 cells were seeded in 24-well tissue culture plates overnight for attachment before transfection. The cells were transfected using Lipofectamine 2000™ transfection reagent according to the manufacturer’s recommendations. Four microliters of Lipofectamine 2000™, 0.8 μg of plasmid DNA (pGL3-Basic [Promega, Madison, WI, USA], pGL3-IFITM1 [−750/−1], pGL3-IFITM1 [−200/−1]), and 0.01 μg of the pRL-CMV Renilla (Promega) were diluted individually in 125-μl aliquots of Gibco Opti-MEM Reduced Serum Medium (Life Technologies). Cells were incubated for 24 h after transfection and treated with IFNα (200 U), and then the luciferase and Renilla activities were measured 24 h later using the Promega Dual-Luciferase Reporter Assay System according to the manufacturer’s instructions. To confirm that the luciferase activity of each construct was caused by IFNα, the activity of each construct was assayed in the presence and absence of IFNα. In a separate set of samples, cells were transfected with the −750/−1 IFITM1 promoter construct for 24 h, followed by transfection with siSTAT1 and siSTAT2 for a further 24 h and subsequent determination of promoter activity. Renilla activity was also assayed to standardize sample transfection efficiencies.

### Statistical analysis

At least three separate experiments were performed for each measurement. All quantitative data were expressed as mean ± standard deviation. Comparisons between two groups were analyzed using *t* tests in Excel 2010 (version 14.0; Microsoft, Redmond, WA, USA), with *P* values less than 0.05 considered statistically significant.

## Results

### IFITM1 is overexpressed in triple-negative SUM149 inflammatory breast cancer cells

**Fig. 1:**
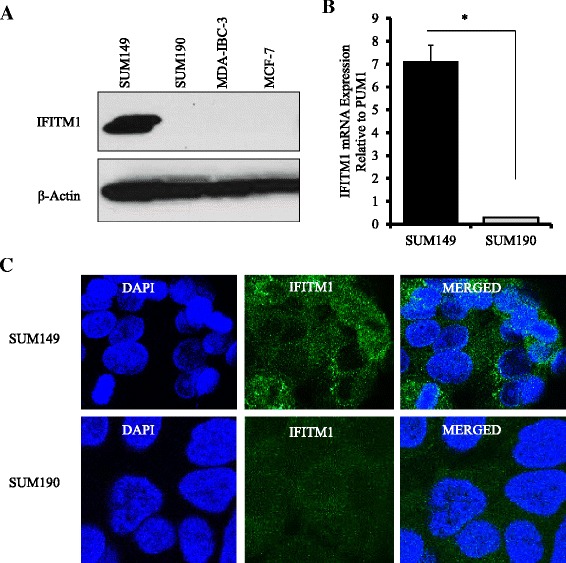
Interferon-induced transmembrane protein 1 (IFITM1) protein and messenger RNA (mRNA) expression in breast cancer cells. **a** IFITM1 protein expression in the triple-negative SUM149 cells, human epidermal growth factor receptor 2–overexpressing SUM190 and MDA-IBC-3 cells, and non–inflammatory breast cancer (non-IBC) MCF-7 cells. The protein expression was assessed by Western blot analysis, with β-actin used as a loading control. **b** IFITM1 mRNA expression in SUM149 and SUM190 cells. The mRNA levels were determined by real-time polymerase chain reaction, and the fold change for each cell line was calculated against the Pumilio RNA-binding family member 1 (PUM1) internal control gene. Each value shown is the mean ± standard deviation from three independent experiments. *P < 0.05. **c** IFITM1 protein expression and localization profiles of SUM149 and SUM190 IBC cells determined by immunofluorescence. The images of the cells were captured using a Leica confocal microscope equipped with Leica Application Suite Advanced Fluorescence Lite 2.6.0 Build 7266 software. *DAPI* 4′,6-diamidino-2-phenylindole

To identify the factors contributing to the aggressive phenotype of IBC cells, we measured the expression of *IFITM1*, an ISG linked to tumor progression, in three IBC cell lines: triple-negative SUM149, HER2-amplified SUM190 and MDA-IBC-3, and the non-IBC cell line MCF-7 (ER+). We found that *IFITM1* was highly expressed in SUM149 cells at the protein (Fig. 1a) and mRNA (Fig. 1b) levels, but that it was not expressed in SUM190, MDA-IBC-3, and MCF-7 cells (Fig. 1a). Immunofluorescence confirmed that IFITM1 was overexpressed in SUM149 cells and that it was localized primarily in the cytoplasmic and plasma membrane regions of the cells (Fig. 1c). We should note that there are only a few human IBC cell lines (i.e., SUM149, SUM190, MDA-IBC-3, MARY-X, and FC-IBC-02) available for studying this complex disease and that these cell lines have molecular signatures that are distinct from each other. Overexpression of *IFITM1* in the SUM149 cell line alone suggests that it might be an important marker in these cells.

### IFITM1 knockdown inhibits proliferation, colony formation, and invasion in SUM149 cells

**Fig. 2:**
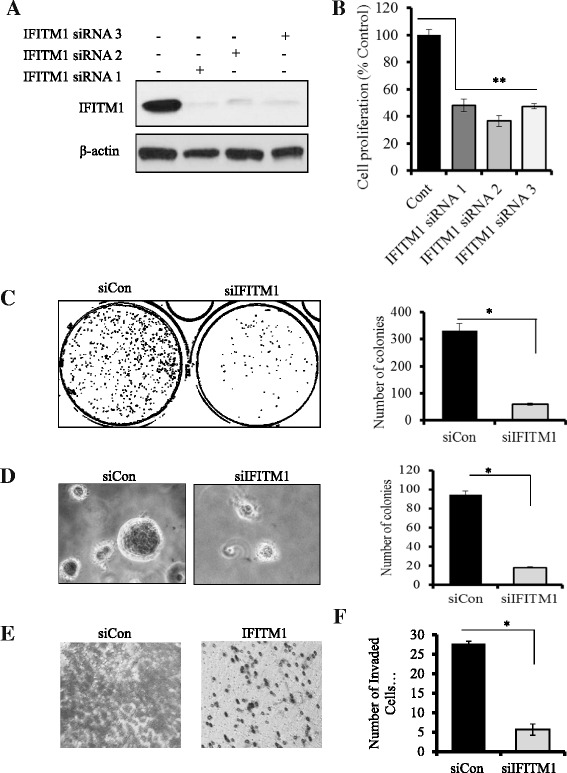
Effects of small interfering RNA (siRNA) knockdown of interferon-induced transmembrane protein 1 (IFITM1) on proliferation and tumorigenic potential of SUM149 cells. **a** Western blot analysis of SUM149 cells showing the protein levels of IFITM1. The IFITM1 gene was knocked down using three separate siRNAs (siRNA 1, siRNA2, and siRNA 3), and the control samples were transfected with a negative control siRNA (siCon) for 72 h. **b** Cell proliferation after 72 h of IFITM1 knockdown with three separate siRNAs. A 3-(4,5-dimethylthiazol-2-yl)-2,5-diphenyltetrazolium bromide assay was performed to assess cell proliferation. Bars represent mean ± standard deviation (SD). **P < 0.005 for siRNA knockdown compared with siCon. **c** 2-D colony formation showing the effects of silencing of IFITM1 in SUM149 cells on the formation of colonies in a 2-D surface. The images of the plates were captured using the ChemiDoc™ XRS System equipped with Image Lab™ software, then transformed and quantified by using ImageJ software. **d** *Left panel*: Anchorage-independent growth in soft agar showing the effect of IFITM1 knockdown on colony formation in SUM149 cells. The representative images were captured using a phase-contrast microscope equipped with an Olympus camera (original magnification, ×200). *Right panel*: The colonies were imaged using the ChemiDoc™ XRS System and quantified using ImageJ software. **e** Effect of IFITM1 knockdown on cell invasion and migration in SUM149 cells as assessed by Transwell Matrigel assay (Corning, Corning, NY, USA). The invaded cells were stained with crystal violet and imaged. **f** Quantitation of the number of invaded cells in SUM149 cells. The data presented are mean ± SD of three replicates. *P < 0.05

**Fig. 3:**
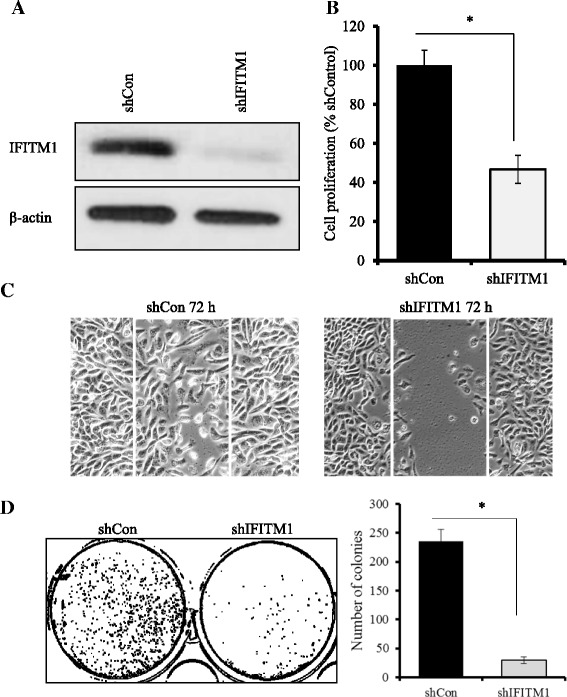
Effects of short hairpin RNA (shRNA) knockdown of interferon-induced transmembrane protein 1 (shIFITM1) on cell proliferation, migration, and colony formation in SUM149 cells. **a** Western blot analysis was used to confirm the shRNA knockdown of IFITM1 in SUM149 cells. **b** Cell proliferation was assessed by 3-(4,5-dimethylthiazol-2-yl)-2,5-diphenyltetrazolium bromide assay in SUM149 cells that were transfected with shRNA for 24 h and maintained in culture for a further 48 h. The assay results showed that the knockdown of IFITM1 reduced cell proliferation by up to 52 % in SUM149 cells compared with the short hairpin control cells (shCon). **c** Cell migration was assessed by wound-healing assay. **d** *Left panel*: 2-D colonies formed by SUM149 cells with and without shRNA knockdown. *Right panel*: Quantification of the number of colonies. *P < 0.05

To assess the functional significance of IFITM1 expression in SUM149 cells, siRNAs and shRNAs were used to knock down IFITM1. Three different siRNAs (IFITM1-siRNA1, IFITM1-siRNA2, and IFITM1-siRNA3) and IFITM1-shRNA were used. Cells were transfected with the individual siRNAs (siRNA-1 and siRNA-2), the pooled siRNA (siRNA-3), shRNA (shIFITM1), scrambled control (siCon), or shRNA control (shCon) for 72 h. We found that the individual siRNAs and the pooled siRNA completely reduced IFITM1 expression in SUM149 cells (Fig. 2a) and that loss of IFITM1 reduced the proliferation of SUM149 cells by approximately 52–63 % (Fig. 2b). Cell-cycle analysis revealed that the inhibitory effect of IFITM1 knockdown in SUM149 cells was due in part to G_1_ arrest (Additional file 1: Figure S1A), which was associated with downregulation of cyclin D1 and cyclin E (Additional file 1: Figure S1B). Additionally, IFITM1 knockdown dramatically reduced the ability of SUM149 cells to form 2-D colonies (Fig. 2c, *left* and *right panels*), to grow in an anchorage-independent manner (Fig. 2d, *left* and *right panels*), and to migrate and invade (Fig. 2e, *left* and *right panels*). We also determined that IFITM1 shRNA completely suppressed IFITM1 expression in SUM149 cells (shown in Fig. 3a), which resulted in inhibition of cell proliferation by up to 54 % (Fig. 3b), migration (Fig. 3c), and 2-D colony formation (Fig. 3d, *left* and *right panels*).

### Enhanced IFNα signaling drives IFITM1 overexpression in SUM149 cells

**Fig. 4:**
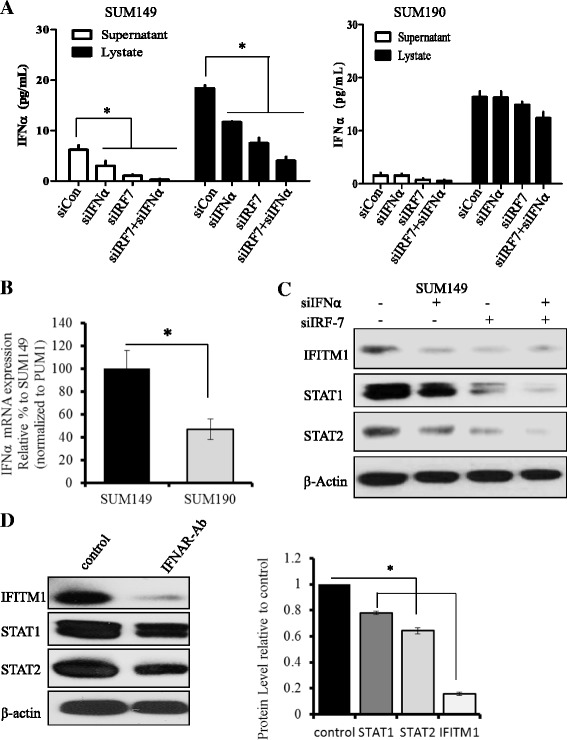
Regulation of interferon-induced transmembrane protein 1 (IFITM1) by the interferon signaling pathway in SUM149 cells. **a** Human interferon α (IFNα) levels were measured in the supernatant and lysates of SUM149 and SUM190 cells after the knockdown of IFNα and interferon regulatory factor 7 (IRF7) using small interfering RNA (siRNA). Measurements of IFNα were done with an enzyme-linked immunosorbent assay (PBL Interferon Source; PBL Assay Science, Piscataway, NJ, USA) according to the manufacturer’s instructions. The data presented are mean ± standard deviation. *P < 0.05 compared with siControl. **b** IFNα messenger RNA (mRNA) levels in SUM149 and SUM190 IBC cells measured by real-time polymerase chain reaction. *P < 0.05. **c** Western blot analysis of IFITM1, signal transducer and activator of transcription 1 (STAT1), and STAT2 protein expression following the suppression of IRF7 and IFNα in SUM149 cells. **d** *Left panel*: Western blot showing the protein levels of IFITM1, STAT1, and STAT2 after interferon receptor (IFNR)-α/β was neutralized using mouse anti-human IFNR-α/β chain 2 monoclonal antibody (Ab) in SUM149 cells. *Right panel*: Quantification of IFITM1 protein expression from the Western blots using ImageJ software. *P < 0.05. *PUM1* Pumilio RNA-binding family member 1

Type I IFNα/β induce ISGs by binding to their receptors IFNAR1/2 and activating the canonical JAK-STAT signaling pathway. To determine whether elevated IFNα signaling is responsible for driving constitutive IFITM1 overexpression in IBC cells, we first measured IFNα levels in SUM149, SUM190, and non-IBC MCF-7 cells using ELISA, and we found that IFNα protein level was significantly higher in the lysates of SUM149 and SUM190 IBC cells compared with non-IBC MCF-7 cells (data not shown). Notably, RT-PCR analysis revealed that IFNα mRNA level was approximately 53 % higher in SUM149 cells compared with SUM190 cells (Fig. 4b). Next, we determined the effect of IFNα suppression in SUM149 and SUM190 cells by transfecting these cells with siRNAs targeting either IFNα and/or its transcriptional regulator IRF7. As shown in Fig. 4a (*left panel*), siRNA knockdown of IFNα significantly reduced IFNα level in the supernatant and lysate of SUM149 cells, whereas knockdown of IRF7, either alone or combined with siIFNα, significantly reduced IFNα level in the lysate and supernatant of SUM149 cells, but the differences were not significant in SUM190 cells (Fig. 4a, *right panel*). Western blot analysis also confirmed that knockdown of IFNα and IRF7, either individually or combined, markedly reduced IFITM1 protein expression in SUM149 cells (Fig. 4c). STAT1 and STAT2 proteins were also reduced following IFNα and IRF7 knockdown (Fig. 4c). Additionally, we found that blockade of the IFNα receptors IFNAR1/2 with a neutralizing monoclonal antibody (IFNAR-Ab) completely inhibited IFITM1 protein expression in SUM149 cells (Fig. 4d, *left* and *right panels*), and it markedly reduced STAT1 and STAT2 protein levels in these cells. Together, these findings confirm that constitutive overexpression of IFITM1 in SUM149 cells is driven in part by activation of the IFNα signaling pathway. Notably, we also performed comparative genomic hybridization (CGH) analysis to assess DNA copy number changes for IFITM1 and other components of the IFN signaling pathway in SUM149 cells. CGH data revealed that there was no significant change in the DNA copy number for IFITM1 in SUM149 cells; however, there was a 27.5 % gain in copy number for IFNα and IFNβ (data not shown), which are the ligands that activate the JAK-STAT signaling pathway. These findings suggest that overexpression of IFITM1 in SUM149 cells may be due in part to increased expression of IFNα (observed at the DNA, mRNA, and protein levels), which activates the JAK-STAT signaling pathway.

### STAT2 is a critical regulator of IFITM1 expression in SUM149 cells

**Fig. 5:**
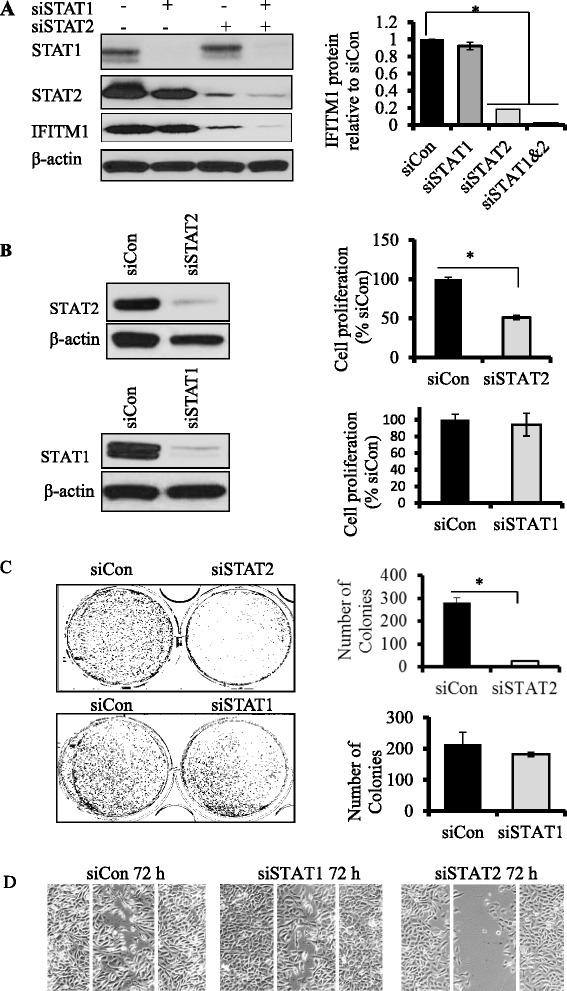
Effects of signal transducer and activator of transcription 1 (STAT1) andSTAT2 knockdown on interferon-induced transmembrane protein 1 (IFITM1) expression and tumorigenic potential of SUM149 cells. **a** *Left panel*: Western blots showing the protein levels of STAT1, STAT2, and IFITM1 after STAT1 and STAT2 genes were knocked down in SUM149 cells using siSTAT1 and siSTAT2. β-Actin was used as a loading control. *Left panel*: Quantification of the protein levels of IFITM1 in the siSTAT1, siSTAT2, and siSTAT1/2 samples. IFITM1 levels were quantified using ImageJ software, were normalized to β-actin, and were expressed relative to siControl. **b** *Upper panel*: Western blot of STAT2 knockdown in SUM149 cells and the effects of the knockdown on cell proliferation. *Lower panel*: Western blot of STAT1 knockdown in SUM149 cells and the effects of the knockdown on cell proliferation. **c** *Left panel*: Effects of STAT1 and STAT2 knockdown on 2-D colony formation in SUM149 cells. *Right panel*: Quantification of the colonies. **d** Wound-healing assay showing the effects of STAT1 and STAT2 knockdowns on the ability of SUM149 cells to migrate. *P < 0.05

STAT1 and STAT2 are transcription factors that play a critical role in regulating type I IFNα/β signaling. To evaluate the role of STAT1 and STAT2 in the regulation of IFITM1 in SUM149 cells, siRNAs were used to knock down their expression. As shown in Fig. 5a (*left* and *right panels*), knockdown of STAT2 completely suppressed IFITM1 expression in SUM149 cells, whereas knockdown of STAT1 did not significantly reduce IFITM1 protein expression in these cells. Similar results were observed using two additional siRNAs targeting STAT1 (siRNA1 and siRNA2) (Additional file 2: Figure S2A) and STAT2 (siRNA1 and siRNA2) (Additional file 2: Figure S2B). Additionally, knockdown of STAT2 reduced cell proliferation (Fig. 5b, *upper left* and *right panels*), colony formation (Fig. 5c, *upper left* and *right panels*), and migration (Fig. 5d, *right panel*) in SUM149 cells, whereas knockdown of STAT1 did not significantly alter the aggressive phenotype of these cells (Fig. 5b and c, *lower left* and *right panels*; Fig. 5d, *middle panel*).

### BRG1 is a critical regulator of IFITM1 expression in SUM149 cells

**Fig. 6:**
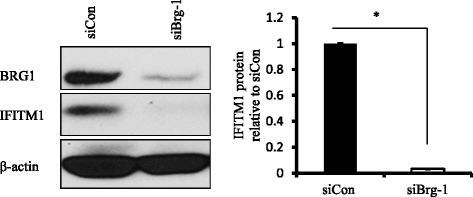
Effects of brahma-related gene 1 (BRG1) knockdown on interferon-induced transmembrane protein 1 (IFITM1) expression in SUM149 cells. *Left panel*: Western blot showing the protein levels of BRG1 and IFITM1 after *BRG1* gene was knocked down using siBRG1 for 48 h in SUM149 cells. β-actin was used as a loading control. *Right panel*: Quantification of IFITM1 protein levels from the Western blots. *P < 0.05

The IFITM1 promoter region has a nucleosome that requires remodeling to expose the interferon-stimulated response element (ISRE), IFNγ-activated site (GAS), and IRF sequences and to enable transcription factor binding. BRG1, the ATP subunit of the BAF chromatin remodeling complex, has been shown to be recruited by STAT2 to the promoter region of ISGs in the process of transcription. To assess the role of BRG1 in regulating IFITM1 expression, siRNA knockdown studies were performed in SUM149 cells, and BRG1 and IFITM1 protein was measured by Western blot analysis. As shown in Fig. 6, siRNA knockdown of BRG1 protein in SUM149 cells completely suppressed IFITM1 expression in these cells. These data suggest that STAT2 and BRG1 are critical regulators of IFITM1 expression in SUM149 cells with less significant involvement of STAT1 in the process.

### STAT2 regulates IFITM1 promoter activity in SUM149 cells

**Fig. 7:**
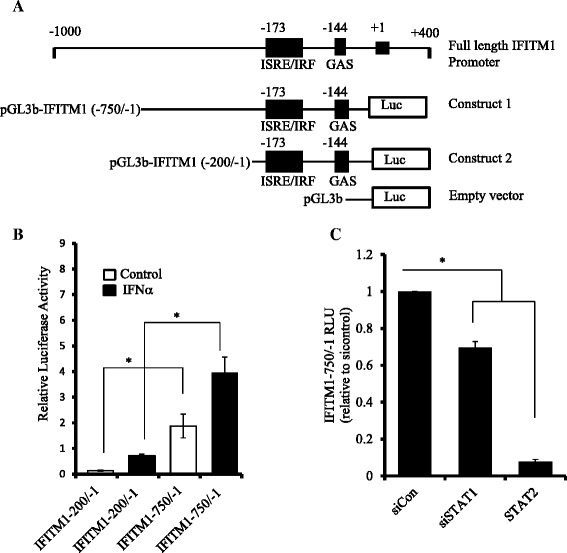
Interferon-induced transmembrane protein 1 (IFITM1) promoter activity in SUM149 cells. **a** Schematic diagram of the IFITM1 promoter showing ATG transcription start site, interferon-stimulated response element (ISRE)/interferon regulatory factor (IRF), interferon gamma-activated site (GAS), TTA half-sites, and TTC half-palindromes; constructs; pGL3-IFITM1 − 750/−1, pGL3-IFITM1 − 200/−1, and the empty vector (pGL3). **b** Relative luciferase activity of the IFITM1 promoter region using the two deletion constructs (−750/−1 and −200/−1), along with interferon treatment in SUM149 cells. **c** Effects of signal transducer and activator of transcription 1 (STAT1) and STAT2 knockdown on the IFITM1 promoter activity using the −750/−1 deletion construct. The luciferase activity was expressed relative to scrambled control RNA (siCon). The data presented are representative of triplicate experiments and are expressed as mean ± standard deviation. *P < 0.05. RLU stands for Relative Light Units (RLU). It is a unit that is based on light emitted by the luciferase-catalyzed chemiluminescent reaction

The promoter region of the IFITM1 gene contains ISRE, GAS, and IRF DNA sequences (Additional file 3: Figure S3) that provide binding sites for transcription factors such as STAT1, STAT2, and IRF9. Figure 7a shows schematic diagrams for the full-length IFITM1 promoter (−1000/+400), the two IFITM1 deletion constructs (IFITM1 − 750/−1 and IFITM1 − 200/−1), and the empty vector (pGL3). A luciferase assay was used to measure IFITM1 promoter activity in SUM149 and SUM190 cells following transfection of these cells with either pGL3b −750/−1 or pGL3b −200/−1. As shown in Fig. 7b, we found that IFITM1 promoter activity was higher in the cells transfected with the −750/−1 construct as compared with −200/−1, and in SUM149 cells as compared with SUM190 cells (data not shown). IFN treatment increased the promoter activity for both constructs significantly in SUM149 cells (Fig. 7b). To determine the functional significance of STAT1 and STAT2 on promoter activity, we knocked down STAT1 and STAT2 in the cells that were transfected with the −750/−1 construct and measured the luciferase activity in the cells. We found that IFITM1 promoter activity was significantly lower in the STAT2 knockdown cells in SUM149 as compared with STAT1 (Fig. 7c), suggesting that STAT2 played a critical role in IFITM1 induction in these cells. STAT2 has been shown to form non–ISG factor 3 (non-ISGF3) complexes in which its homodimers bind to GAS consensus sequence on the promoters of a subset of ISGs and induce their transcription. Of the two constructs, the −750/−1 construct has more TTA half-sites and TTC half-palindromes compared with the −200/−1 construct, which may explain why it had higher luciferase activity. Taken together, these findings suggest that STAT2, not STAT1, plays a dominant role in regulating IFITM1 transcriptional activation in SUM149 cells through binding to multiple consensus sequences such as ISRE/IRF and GAS.

### Functional integrity of the IFNα signaling pathway in SUM149 cells

**Fig. 8:**
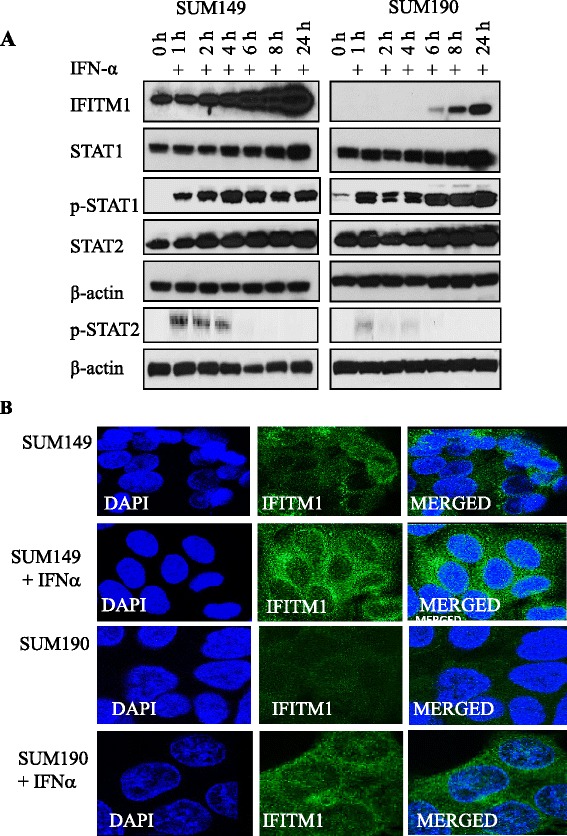
Activation of type I interferon (IFN) signaling pathway by exogenous IFNα in SUM149 and SUM190 cells. **a** Effects of IFN induction on the protein levels of IFN-stimulated genes (IFITM1, STAT1, p-STAT1, STAT2, and p-STAT2) in the SUM149 and SUM190 cells. β-Actin was used as a loading control. **b** Cellular localization and expression of interferon-induced transmembrane protein 1 (IFITM1) protein in SUM149 and SUM190 cells following IFN induction. Immunofluorescence was used to assess IFITM1 expression and localization in the cells that were induced with IFNα for 24 h as compared with the control. The images were captured using Leica confocal microscope. *DAPI* 4′,6-diamidino-2-phenylindole, *STAT* signal transducer and activator of transcription

Because IFITM1 was constitutively overexpressed in SUM149 cells, we next determined whether exogenous IFNα was capable of activating the IFNα signaling pathway in these cells. As shown in Fig. 8a, exogenous treatment with IFNα increased IFITM1 protein expression in both SUM149 and SUM190 cells within 6–8 h of treatment, with maximum induction at 24 h; however, basal expression of IFITM1 was observed in SUM149 cells from time 0 but not in SUM190 cells until 6 h posttreatment with IFNα. Notably, exogenous IFNα also increased total STAT1 and STAT2 protein expression in both cell lines, but the induction of STAT1 and STAT2 by exogenous IFNα was less robust than that of IFITM1. Interestingly, p-STAT2 (Tyr690) was induced significantly more in SUM149 than in SUM190 cells 1 h after treatment with IFNα and faded after 5 h. The higher STAT2 phosphorylation in SUM149 cells further supports the critical role it plays in IFITM1 overexpression and partly explains the differences in IFITM1 expression in the IBC cell lines. Additionally, immunofluorescence data (Fig. 8b) showed that exogenous addition of IFNα increased IFITM1 protein expression in both IBC cell lines. Overall, these results show that type I IFN signaling is constitutively enhanced in SUM149 cells; however, these cells are still responsive to exogenous IFNα.

## Discussion

IBC is a rare and highly aggressive subtype of breast cancer that is not well characterized at the molecular level. In this study, we report a novel role for the ISG *IFITM1* in promoting the aggressive phenotype of SUM149 IBC cells. In particular, we show that IFITM1 was constitutively overexpressed at the mRNA and protein levels in triple-negative SUM149 cells but was not expressed in HER2-amplified SUM190 or MDA-IBC-3 IBC cells. We also found that IFITM1 overexpression promoted—whereas its knockdown inhibited—proliferation, migration/invasion, and tumorigenicity in vitro*.* Furthermore, we demonstrate that SUM149 cells expressed and secreted elevated levels of type I IFNα and that blockade of IFNα signaling using a neutralizing antibody against its receptor, IFNAR1/2, or knockdown of STAT2, suppressed IFITM1 expression in SUM149 cells. Loss of *IFITM1* expression dramatically reduced the ability of these cells to proliferate, migrate, invade, and form colonies in soft agar. We should note that differential regulation of IFN response genes has been observed in many human malignancies, including leukemia [21], ovarian cancer [23], gastric cancer [14], lung cancer [24], colon cancer [25], and breast cancer [26] and that high expression of ISGs is associated with poor clinical outcome [14]. However, to our knowledge, this study is the first to highlight a critical role for the IFNα signaling pathway and the IFN response gene *IFITM1* in enhancing the aggressive phenotype of triple-negative SUM149 cells. The fact that IFITM1 was highly expressed in SUM149 cells, which are triple-negative, but was not expressed in HER2-amplified SUM190 and MDA-IBC-3 cells suggests that it might be a unique molecular marker of TNIBC and that it could be a potential therapeutic target in patients with TNIBC. Approximately 60 % of IBCs are basal-like or triple-negative, as characterized by the ER−, PR−, and human epidermal growth factor receptor 2 (HER2)-nonamplified status [27]. Patients with TNBC have a lower overall survival rate [28] and very limited therapeutic options. In contrast, HER2 amplification occurs in approximately 40 % of IBCs [29, 30], and IBC patients with HER2-amplified tumors have been shown to respond favorably to anti-HER2 therapies [31].

Over the past 3 decades, IFNs (α, β, γ) have established a reputation for being immunologic guardians against disease and as promising antitumor agents [32–35]; however, recent evidence suggests that IFNs may also promote tumor progression. Indeed, it has been reported that breast cancer patients whose tumors express high IFN response genes are 1.7 times more likely to develop metastasis and to die as a result of the disease as compared with patients whose tumors express low levels of the IFN response gene signature [36]. Furthermore, increased expression of a subset of ISGs, including *IFITM1*, *EIF2AK2*, *STAT1*, and *IFI27*, has been reported in several types of cancers, and these ISGs have been shown to promote tumor growth and resistance to chemotherapy and radiotherapy [11, 37, 38]. Consistent with these reports, our present study revealed constitutive activation of the IFNα signaling pathway in IBC cells, which was strongly associated with IFITM1 overexpression. Notably, blockade of IFNα signaling using a neutralizing antibody against the type I IFN receptor, IFNAR1/2, completely suppressed IFITM1 expression, and it markedly reduced the tumorigenic potential of SUM149 cells in vitro*.* While the mechanism by which IFITM1 overexpression enhances the aggressiveness of SUM149 cells is not known, we should note that Ras homolog gene family member C GTPase (RhoC-GTPase) is overexpressed in 90 % of IBC tumors as compared with 38 % of the stage-matched non-IBC tumors and that in SUM149 cells overexpression of RhoC-GTPase is associated with loss of WNT1 inducible signaling pathway 3 (WISP3). Conversely, restoration of WISP3 downregulates RhoC-GTPase and inhibits the invasive potential of SUM149 cells [39]. Indeed, RhoC-GTPase is found to play an essential role in the metastatic behavior of IBC by increasing all aspects of metastatic process, such as cellular motility and invasion, cytoskeletal assembly, and cell adhesion. RhoC-GTPase controls the cytoskeletal reorganization by inducing actin stress fiber and focal adhesion contact formation [40, 41]. While the potential interaction between IFITM1 and RhoC-GTPase was not directly assessed in our study, it has been reported that Rho-GTPases can interact with caveolin 1 (CAV-1) in cancer cells [42]. Notably, IFITM1 has recently been shown to interact with CAV-1 in colorectal cancer cells, and this interaction impacts the ability of colorectal cancer cells to migrate and invade [43]. Additionally, IFITM1 has also been shown to enhance migration and invasion in head and neck cancer cells through activation of matrix metalloproteinase 12 (MMP12) and MMP13, key enzymes involved in the degradation of the basement membrane that allows cells to infiltrate into adjacent tissues [15].

IFNα and IFNβ are produced by many cell types, including plasmacytoid dendritic cells, lymphocytes, macrophages, and fibroblasts; however, there is evidence that IFNs can also be produced by tumor cells themselves [44–46]. Elevated levels of IFN have been reported in cancer cells as compared with normal primary cells or normal tissues [18, 47, 48]. Furthermore, increased expression of some IFN-induced genes has been shown to be higher in metastatic cancer cells than in nonmetastatic cells [49]. Our data suggest that IFNα levels are significantly elevated in IBC cells as compared with non-IBC cells and that blockade of IFN signaling in these cells markedly reduces IFITM1 expression. It is critical to note that IRF3 and IRF7 are the key regulators of type I IFN production and thus play a central role in innate immunity [50]. IRF7 regulates the transcription of IFNα/β genes and ISGs by binding to an ISRE in their promoters via the virus-activated, myeloid differentiation primary response gene 88 (MyD88)-independent pathway and the Toll-like receptor-activated, MyD88-dependent pathway. Notably, we found that IRF7 was markedly elevated (>2.5-fold) at the mRNA and protein levels in SUM149 cells compared with SUM190 cells (data not shown) and that knockdown of IRF7 reduced IFNα level and blocked its signaling through IFNAR1/2 (Fig. 4a). Furthermore, we found that suppression of IFNα and IRF7 dramatically reduced IFITM1 expression in SUM149 cells (Fig. 4b), thus confirming the importance of the IFNα signaling pathway in driving IFITM1 expression in these cells. We should note that while endogenous IFNα was elevated in the IBC cells, exogenous addition of IFNα was still able to induce IFITM1, STAT1, and STAT2 in these cells; however, the further induction of these ISGs in SUM149 cells did not alter the growth or aggressive phenotype of these cells.

**Fig. 9:**
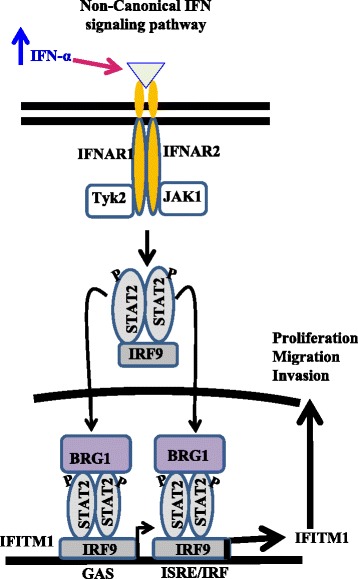
Schematic diagram depicting the proposed signaling pathway involved in interferon-induced transmembrane protein 1 (IFITM1) upregulation in SUM149 cells. IFITM1 is one of the interferon-stimulated genes (ISGs) that are induced through the canonical Janus kinase/signal transducer and activator of transcription (JAK-STAT) pathway due to increased expression of interferon α (IFNα). The binding of IFNα to its receptor interferon (alpha, beta and omega) receptor (IFNAR1/2) leads to the induction of the canonical JAK-STAT pathway, which involves phosphorylation of STAT1, STAT2, and the formation of the complex interferon-stimulated gene factor 3 (ISGF3), ultimately inducing many ISGs, including IFITM1. In the nucleus, ISGF3 recruits chromatin remodeling complex brahma-related gene 1 (BRG1) via STAT2 to remodel the promoter and expose interferon-stimulated response element (ISRE)/interferon gamma-activated site (GAS) for transcription factor binding. Alternatively, STAT2 can homodimerize upon phosphorylation, and bind interferon regulatory factor 9 (IRF9) to form a non-ISGF3 complex that is capable of binding the GAS or ISRE/IRF sequences at the promoter region of a subset of ISGs and induce their transcription without the participation of STAT1 in a noncanonical interferon signaling pathway. The presence of both GAS and ISRE/IRF consensus sequences at the IFITM1 promoter suggests that its transcription can be induced by both canonical and noncanonical signaling pathways, resulting in its overexpression in SUM149 cells

One of the most prominent findings of our study was the identification of STAT2 as the critical regulator of IFITM1 expression in SUM149 cells. The role of STAT2 in canonical JAK-STAT signaling as a part of ISGF3 complex is well documented; however, there is strong evidence that STAT2 can also homodimerize and combine with IRF9 to form an ISGF3-like complex [51] that translocates to the nucleus. Once in the nucleus, STAT2 recruits BRG1 to the complex, which then binds to the ISRE or GAS sequence of IFITM1 promoter and induces its transcription through noncanonical IFN signaling. It has been reported that STAT2 complexes, without STAT1, are capable of inducing a subset of ISGs without the formation of ISGF3 [52, 53]. For instance, STAT2 was reported to mediate STAT1-independent protection against dengue virus infection in mice that were deficient in STAT1 through the formation of non-ISGF3 complexes that involved STAT2 homodimers, and did not require STAT1 [54]. Additionally, Brierley et al. [52, 55] reported that STAT2 was critical for induction of GAS-regulated target genes, which was independent of ISGF3. Our study shows that knockdown of STAT2, but not STAT1, completely reduced IFITM1 expression and promoter activity in SUM149 cells. We also found that knockdown of BRG1, the chromatin remodeling protein, also completely suppressed IFITM1 expression in SUM149 cells. BRG1 is the ATP subunit of the chromatin remodeling complex BAF, and studies have shown that STAT2 and BRG1 interact to induce the expression of certain ISGs, including IFITM1, through noncanonical IFN signaling [56, 57]. There is also evidence that the recruitment of BAF to the IFITM1 promoter is mediated in part by the interaction of BRG1 with STAT2 [58–60]. In our working model shown in Fig. 9, we propose that overexpression of IFITM1 in SUM149 cells is driven by increased expression of IFNα, which activates the noncanonical type I IFN signaling pathway where STAT2 homodimers recruit BRG1 to remodel the promoter and expose ISRE, GAS, and IRF consensus sequences for the binding of transcription factors. This model is supported by a study conducted by Blaszczyk et al. [51], which showed that IFNα induction leads to formation of both ISGF3 and STAT2/IRF9 complexes and that the induction of ISGs due to ISGF3 was rapid and transient, while that due to STAT2/IRF9 was slow and prolonged, leading to the expression of ISGs long after the initial induction had ended. Whereas other ISGs have either the ISRE or the GAS sequences, the IFITM1 promoter region has both the ISRE and GAS sequences as well as IRF sequences (Additional file 3: Figure S3). Additionally, STAT2 knockdown in SUM149 cells yielded results similar to those for IFITM1 knockdown; however, STAT1 knockdown had little or no effect on the growth or invasive potential of these cells, thus supporting a critical role for STAT2-BRG1 crosstalk in regulating IFITM1 expression in these cells.

## Conclusions

Our present study reveals a critical role for the IFNα signaling pathway and IFITM1 overexpression in promoting the aggressive phenotype of the TNIBC cell line SUM149. The fact that IFITM1 is not expressed in the HER2-amplified IBC cell lines SUM190 and MDA-IBC-3 suggests that IFITM1 overexpression might be a unique marker of aggressiveness in TNIBC. However, further studies using additional TNIBC cell lines and IBC tumors are needed to fully assess the prognostic and therapeutic potential of IFITM1 expression in IBC.

## Additional files

Additional file 1: Figure S1. Effects of IFITM1 knockdown on cell cycle and cell-cycle proteins in SUM149 cells. (PPT 212 kb) Additional file 2: Figure S2. STAT1 and STAT2 knockdown in SUM149 using three different siRNAs. (PPT 185 kb) Additional file 3: Figure S3. IFITM1 promoter sequence. (PPT 78 kb)

## Abbreviations

ATP: adenosine triphosphate
BAF: brahma-associated factor
BRG1: brahma-related gene 1
CAV-1: caveolin 1
cDNA: complementary DNA
CGH: comparative genomic hybridization
DAPI: 4′,6-diamidino-2-phenylindole
ELISA: enzyme-linked immunosorbent assay
ER: estrogen receptor
FBS: fetal bovine serum
GAS: interferon gamma-activated site
HER2: human epidermal growth factor receptor 2
IBC: inflammatory breast cancer
IFI27: interferon-inducible protein 27
IFIT1: interferon-induced protein with tetratricopeptide repeats 1
IFITM1: interferon-induced transmembrane protein 1
IFN: interferon
IFNAR: interferon (alpha, beta and omega) receptor
IgG: immunoglobulin G
IRF: interferon regulatory factor
ISG: interferon-stimulated gene
ISGF3: interferon-stimulated gene factor 3
ISRE: interferon-stimulated response element
JAK: Janus kinase
MMP: matrix metalloproteinase
mRNA: messenger RNA
MTT: 3-(4,5-dimethylthiazol-2-yl)-2,5-diphenyltetrazolium bromide
MyD88: myeloid differentiation primary response gene 88
PBS: phosphate-buffered saline
PLSCR1: phospholipid scramblase 1
PUM1: Pumilio RNA-binding family member 1
RhoC-GTPase: Ras homolog gene family, member C GTPase
RIPA: radioimmunoprecipitation assay
RLU: Relative Light Units
RT-PCR: real-time polymerase chain reaction
SD: standard deviation
SDS: sodium dodecyl sulfate
shRNA: short hairpin RNA
siCon: scrambled control RNA
siIFN: small interfering interferon
siRNA: small interfering RNA
STAT: signal transducer and activator of transcription
TBS-T: Tris-buffered saline and Tween 20
TNIBC: triple-negative inflammatory breast cancer
WISP3: WNT1 inducible signaling pathway 3
