# Supplementary material for: Interferon-induced transmembrane protein 1 (IFITM1) overexpression enhances the aggressive phenotype of SUM149 inflammatory breast cancer cells in a signal transducer and activator of transcription 2 (STAT2)-dependent manner
Source: Breast Cancer Res. 2016 Feb 20;18:25. doi: 10.1186/s13058-016-0683-7 (PMC4761146; doi:10.1186/s13058-016-0683-7)
Supplement: Additional file 1: Figure S1. — Effects of IFITM1 knockdown on cell cycle and cell-cycle proteins in SUM149 cells. (PPT 212 kb) [file 13058_2016_683_MOESM1_ESM.ppt]

## Slide 1
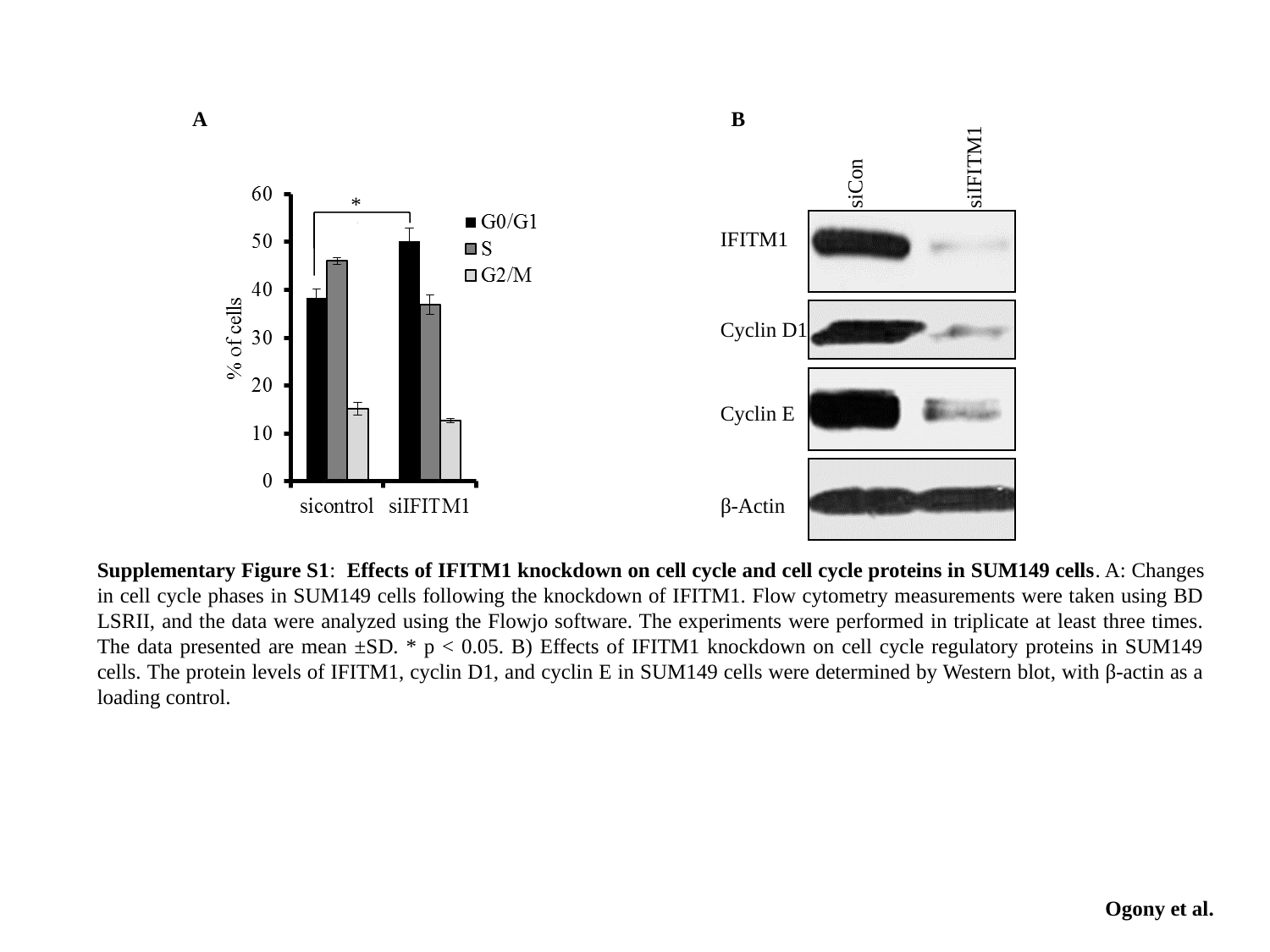

A
B
siIFITM1
siCon
*
IFITM1
Cyclin D1
Cyclin E
β-Actin
Supplementary Figure S1: Effects of IFITM1 knockdown on cell cycle and cell cycle proteins in SUM149 cells. A: Changes in cell cycle phases in SUM149 cells following the knockdown of IFITM1. Flow cytometry measurements were taken using BD LSRII, and the data were analyzed using the Flowjo software. The experiments were performed in triplicate at least three times. The data presented are mean ±SD. * p < 0.05. B) Effects of IFITM1 knockdown on cell cycle regulatory proteins in SUM149 cells. The protein levels of IFITM1, cyclin D1, and cyclin E in SUM149 cells were determined by Western blot, with β-actin as a loading control.
Ogony et al.
