# Supplementary material for: Interferon-induced transmembrane protein 1 (IFITM1) overexpression enhances the aggressive phenotype of SUM149 inflammatory breast cancer cells in a signal transducer and activator of transcription 2 (STAT2)-dependent manner
Source: Breast Cancer Res. 2016 Feb 20;18:25. doi: 10.1186/s13058-016-0683-7 (PMC4761146; doi:10.1186/s13058-016-0683-7)
Supplement: Additional file 2: Figure S2. — STAT1 and STAT2 knockdown in SUM149 using three different siRNAs. (PPT 185 kb) [file 13058_2016_683_MOESM2_ESM.ppt]

## Slide 1
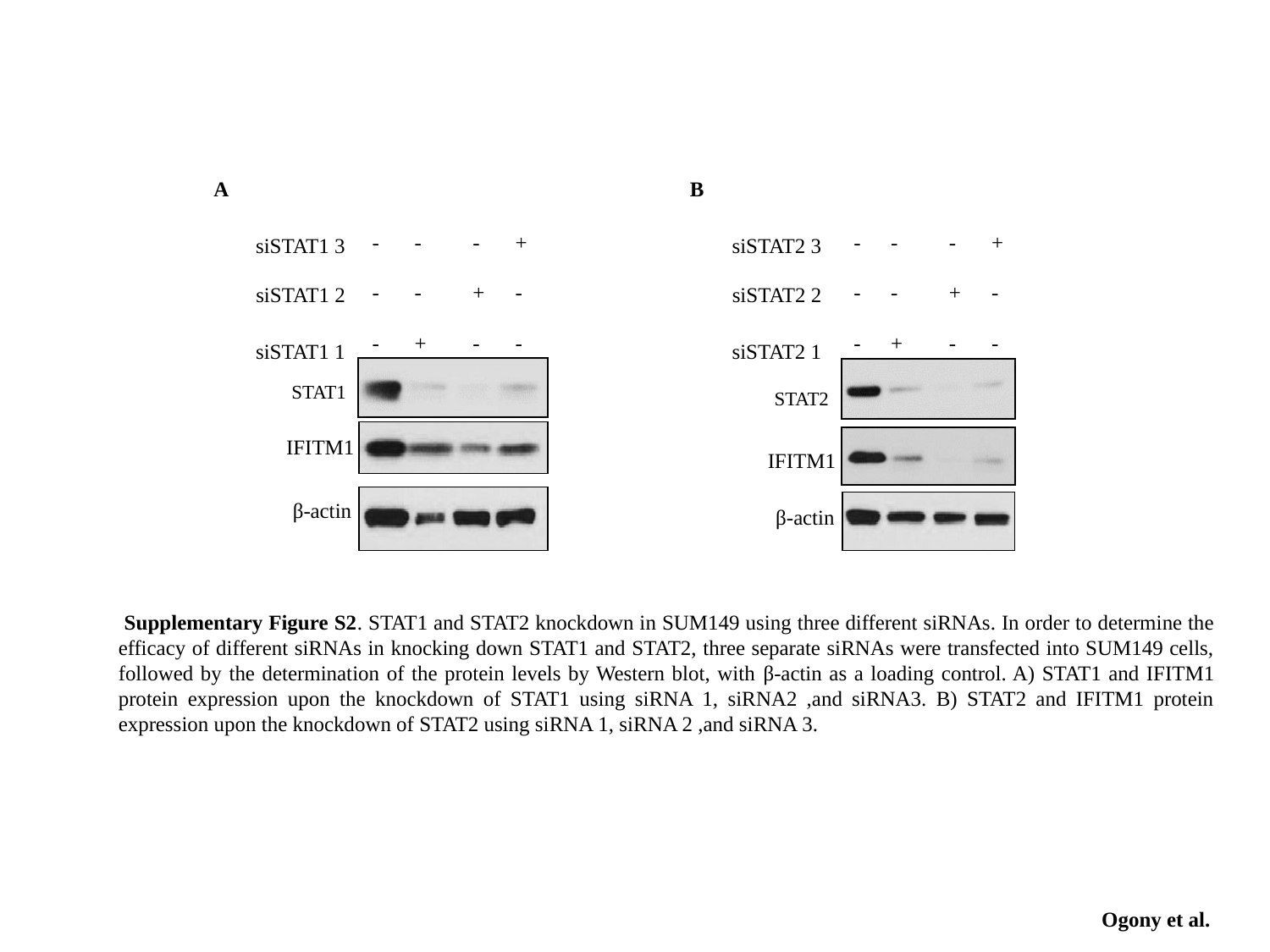

A
B
-
-
-
-
-
+
-
+
-
+
-
-
siSTAT1 3
siSTAT1 2
siSTAT1 1
STAT1
IFITM1
β-actin
-
-
-
-
-
+
-
+
-
+
-
-
siSTAT2 3
siSTAT2 2
siSTAT2 1
STAT2
IFITM1
β-actin
 Supplementary Figure S2. STAT1 and STAT2 knockdown in SUM149 using three different siRNAs. In order to determine the efficacy of different siRNAs in knocking down STAT1 and STAT2, three separate siRNAs were transfected into SUM149 cells, followed by the determination of the protein levels by Western blot, with β-actin as a loading control. A) STAT1 and IFITM1 protein expression upon the knockdown of STAT1 using siRNA 1, siRNA2 ,and siRNA3. B) STAT2 and IFITM1 protein expression upon the knockdown of STAT2 using siRNA 1, siRNA 2 ,and siRNA 3.
Ogony et al.
