# Supplementary material for: Interferon-induced transmembrane protein 1 (IFITM1) overexpression enhances the aggressive phenotype of SUM149 inflammatory breast cancer cells in a signal transducer and activator of transcription 2 (STAT2)-dependent manner
Source: Breast Cancer Res. 2016 Feb 20;18:25. doi: 10.1186/s13058-016-0683-7 (PMC4761146; doi:10.1186/s13058-016-0683-7)
Supplement: Additional file 3: Figure S3. — IFITM1 promoter sequence. (PPT 78 kb) [file 13058_2016_683_MOESM3_ESM.ppt]

## Slide 1
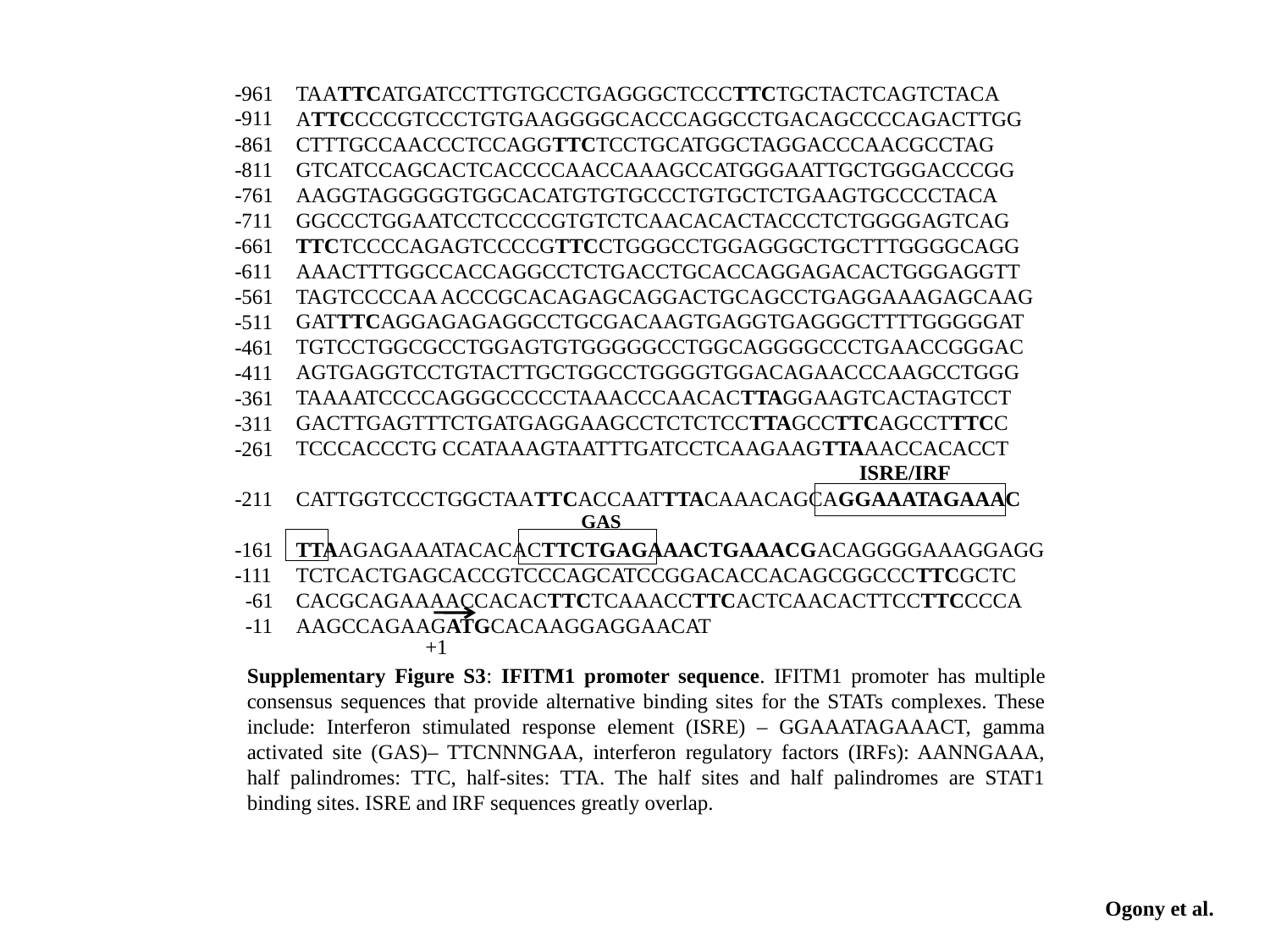

-961
TAATTCATGATCCTTGTGCCTGAGGGCTCCCTTCTGCTACTCAGTCTACA
ATTCCCCGTCCCTGTGAAGGGGCACCCAGGCCTGACAGCCCCAGACTTGG
CTTTGCCAACCCTCCAGGTTCTCCTGCATGGCTAGGACCCAACGCCTAG
GTCATCCAGCACTCACCCCAACCAAAGCCATGGGAATTGCTGGGACCCGG
AAGGTAGGGGGTGGCACATGTGTGCCCTGTGCTCTGAAGTGCCCCTACA
GGCCCTGGAATCCTCCCCGTGTCTCAACACACTACCCTCTGGGGAGTCAG
TTCTCCCCAGAGTCCCCGTTCCTGGGCCTGGAGGGCTGCTTTGGGGCAGG
AAACTTTGGCCACCAGGCCTCTGACCTGCACCAGGAGACACTGGGAGGTT
TAGTCCCCAA ACCCGCACAGAGCAGGACTGCAGCCTGAGGAAAGAGCAAG
GATTTCAGGAGAGAGGCCTGCGACAAGTGAGGTGAGGGCTTTTGGGGGAT
TGTCCTGGCGCCTGGAGTGTGGGGGCCTGGCAGGGGCCCTGAACCGGGAC
AGTGAGGTCCTGTACTTGCTGGCCTGGGGTGGACAGAACCCAAGCCTGGG
TAAAATCCCCAGGGCCCCCTAAACCCAACACTTAGGAAGTCACTAGTCCT
GACTTGAGTTTCTGATGAGGAAGCCTCTCTCCTTAGCCTTCAGCCTTTCC
TCCCACCCTG CCATAAAGTAATTTGATCCTCAAGAAGTTAAACCACACCT
CATTGGTCCCTGGCTAATTCACCAATTTACAAACAGCAGGAAATAGAAAC
TTAAGAGAAATACACACTTCTGAGAAACTGAAACGACAGGGGAAAGGAGG
TCTCACTGAGCACCGTCCCAGCATCCGGACACCACAGCGGCCCTTCGCTC
CACGCAGAAAACCACACTTCTCAAACCTTCACTCAACACTTCCTTCCCCA
AAGCCAGAAGATGCACAAGGAGGAACAT
-911
-861
-811
-761
-711
-661
-611
-561
-511
-461
-411
-361
-311
-261
ISRE/IRF
-211
GAS
-161
-111
-61
-11
+1
Supplementary Figure S3: IFITM1 promoter sequence. IFITM1 promoter has multiple consensus sequences that provide alternative binding sites for the STATs complexes. These include: Interferon stimulated response element (ISRE) – GGAAATAGAAACT, gamma activated site (GAS)– TTCNNNGAA, interferon regulatory factors (IRFs): AANNGAAA, half palindromes: TTC, half-sites: TTA. The half sites and half palindromes are STAT1 binding sites. ISRE and IRF sequences greatly overlap.
Ogony et al.
